# Koch on Cholera

**Published:** 1894-09-29

**Authors:** 


### The Hospital

AN INSTITUTIONAL JOURNAL OF

### The Medical Sciences and Hospital Administration,

WITH

AN EXTRA NURSING SUPPLEMENT.

VOL. XVI.—No. 418.] [SEPT. 29, 1894.

### KOCH ON CHOLERA.

DR. KOCH reminds the members of the medical profession that cholera, when once it has taken possession of Europe, has not usually, in times past, been expelled in less than ten years. Only three years have passed since the commencement of the present invasion. We have, therefore, still seven years of grave possibilities to look forward to.

There are many people who refuse to take note of danger, except when it is forcibly thrust into their own immediate presence. Such people Dr. Koch would like to send to Niedzwedzen, on the Russian frontier. There has been an outbreak of cholera at that devoted spot, and as many as 13 per cent. of the whole population have fallen victims to the disease. All Europe, not excluding Great Britain, may consider itself as now actually invaded by the deadliest pestilence known to modern medicine. If we consider the extraordinary speed with which the infection sometimes moves, and the absolute uncertainty of the direction which the epidemic will take, it is obvious that the most elementary prudence dictates to all European communities the necessity for being ready at any moment to take up a position of hygienic siege. Dr. Koch has definitely assumed the attitude of a hygienic general. He took the opportunity, at the Public Health Congress at Magdeburg the other day, of recounting the dangers of the European situation, surveying the forces available for dealing with possible epidemics, and laying down such general principles of action as comport with modern medical ideas.

Those medical men who belong to the class of independent thinkers will read Dr. Koch's utterances with sincere approval. He speaks like a man who has observed widely and considered soberly the manifold facts and difficulties of the case to be dealt with. On the question of the actual cause of cholera he is of course the protagonist of the bacillus theory. But he does not permit the theory to ride off with him, as too many of his impulsive disciples do. "We all agree," says he, "that a certain parasite is the cause of cholera. But we must also take into consideration a great number of secondary causes, such as the local, temporal, and personal conditions which have come to the aid of such a parasite in order to bring about a case of the disease." There speaks the true man of science and of the world. Of all the enemies which a progressive science has to dread not one is more injurious to true progress than the mere scientific faddist, the man who makes a new discovery once a week, and who insists that every one of his "discoveries" shall be entered upon the national statute book, with penal clauses for every man who refuses to put them into practice in actual life. Dr. Koch recognises that the possessors of the new medical knowledge of the times incur responsibilities to their various states which can only be properly discharged by wisdom of the soberest, and that mental breadth which the statesman is compelled to cultivate.

The inexperienced scientific faddist, perceiving, as he supposes, the striking success of the protective inoculations against cholera, forthwith demands general inoculation, and treats with airy contempt the old-fashioned preventionist who purifies the drinking water and insists upon all other necessary conditions for the maintenance of the general health. What is this but saying that in the case of a foreign invasion it is of no use to have a first line of defence or a second line of defence; that the only wise method is for everybody to betake himself to the citadel and there to await the onslaught of the enemy, secure in armour of impenetrable mail? Dr. Koch recognises the folly, and worse than folly, of all such unscientific enthusiasm. He sees, as every practical man sees, that the more lines of defence we have, provided the defences are really defensive, the better it will be for us, and that no country is so safe and so happy as that country which keeps a foreign army or a deadly disease absolutely outside the very outermost of its defences. Let us grant that protective inoculation against cholera will prove to be everything which its disciples claim that it is, we must still feel that no soldier is so likely to escape without wounds as he who never comes within sight of the enemy. Let us inoculate and protect ourselves by all means; but let us consider ourselves most of all happy when our outermost sanitary defences are so strong and so excellent that we do not need to put the merits of our protective inoculation to the proof.

It need not have caused any surprise if Dr. Koch, who has risen to fame by the excellence of his laboratory work, had insisted upon the purely bacteriological aspects of national cholera defences. But he has done otherwise, and in taking the stand he has taken he has subordinated the mere specialist to the medical statesman, and has thereby earned the rational confidence, not only of his fellow countrymen, but of thoughtful physicians and statesmen everywhere. He tells his *confrères* of the old and familiar measures which have been taken under his direction for the preservation of the German Empire from cholera. "The measures which have been taken to combat infection," he declares, "I do not hesitate to say, have been successful." This declaration is easily put to the proof. That part of Germany over which Dr. Koch has medical authority was the scene of a spreading cholera epidemic three years ago, and to a less extent two years ago. In proportion as preventive measures have been strictly employed has the epidemic been effectually subdued. That these conditions stand to each other in the relation of cause and effect is practically proved by the fact that in neighbouring countries, and more especially in Russia, where similar measures have not been extensively and thoroughly employed, no similar subsidence of cholera has taken place. On the contrary, as at Niedzwedzen, whole populations have been attacked, and have died by hundreds, and the forces of death still march grimly on.

The sanitary leaders of our own country are universally of Dr. Koch's mind. Whilst indulging the hope that protective inoculation against cholera may prove an efficient inner line or citadel of defence if the worst should come to the worst, they still know and act upon the knowledge that the deadlier the enemy the greater is the distance he should be kept at. All our younger scientists may well learn a practical lesson from Koch, and so, too, may the press and the public. During the past decade the more youthful portion of the profession of medicine has appeared to many to resemble nothing so much as an assemblage of conjurors, in which each has vied with all the rest in the production of new and startling feats of legerdemain. This disease is to be cured by a *tour de force*, that is, to be carried at the point of the bayonet, and the world is to be constantly astonished by an exhibition of medical miracles in comparison with which the signs and wonders of the Roman Church are as mere child's play. This is not science and it is not sense. But it is very injurious to true medical progress.

It is a pity that the physiological laboratory is almost exclusively occupied by young men, and men who are bent upon making reputations which shall hereafter turn to financial profit. We are not blaming the men. Their ambitions are not only natural, but universal. But for all that, the man of science who follows science in order that science may make him rich can seldom add any serious and worthy contribution to the great fabric of true and permanent knowledge. Older men, more responsible men, men of wide general knowledge, are needed in the fields of scientific medicine. The man who sees the splendid possibilities of modern medical science grieves much at the public and private indifference with which those possibilities are regarded. Prosperous private munificence might open permanent careers for scores of competent young scientists who will be forced by the poverty or non-existence of scientific endowments into the more profitable craft of medical practice against both their tastes and their judgment. But they are convinced by the resistless logic of the need for bread and butter. Even the State, which now and then buys a costly picture, might give an occasional glance in the direction of the endowment of medical research and solid progress. What is wanted is that men shall be kept at science until their powers are matured and their minds are broadened with the breadth of ripened manhood. Then we shall have true progress instead of mere startling wonders of crude speculation which are born and die in a day.

